# Guillaine-barre syndrome; a rare complication of melioidosis. a case report

**DOI:** 10.1186/s12879-016-1719-4

**Published:** 2016-08-09

**Authors:** P. W. M. C. S. B. Wijekoon, K. A. S. Bandara, A. Kailainathan, N. S. Chandrasiri, C. T. Hapuarachchi

**Affiliations:** 1 Faculty of Medical Sciences, University of Sri Jayewardenepura, Nugegoda, Sri Lanka; 2 Colombo South Teaching Hospital, Kalubowila, Dehiwala-Mount Lavinia, Sri Lanka

**Keywords:** Melioidosis, Guillain barre syndrome, Plasmapheresis, Case report, Sri Lanka

## Abstract

**Background:**

Melioidosis caused by *Burkholderia pseudomellei* is an infection with protean clinical manifestations. Guillain-Barré syndrome [GBS] associated with melioidosis is very rare.

**Case presentation:**

A 42-year-old woman with diabetes presented with abdominal pain, vomiting and intermittent fever for one month. Six months before presentation she had recurrent skin abscesses. Three months before presentation she had multiple liver abscesses which were aspirated in a local hospital. The aspirate grew “coliforms” resistant to gentamicin and sensitive to ceftazidime.

On presentation she had high fever and tender hepatomegaly. Ultra Sound Scan of abdomen showed multiple liver and splenic abscesses. Based on the suggestive history and sensitivity pattern of the previous growth melioidosis was suspected and high dose meropenem was started. Antibodies to melioidin were raised at a titre of 1:10240. The growth from the aspirate of liver abscess was confirmed as *Burkholderia pseudomellei* by polymerase chain reaction [PCR].

After a week of treatment, patient developed bilateral lower limb weakness. Deep tendon reflexes were absent. There was no sensory loss or bladder/bowel involvement. Analysis of the cerebro-spinal fluid showed elevated proteins with no cells. There was severe peripheral neuropathy with axonal degeneration. A diagnosis of GBS was made and she was treated with plasmapharesis with marked improvement of neurological deficit.

Continuation of intravenous antibiotics lead to further clinical improvement with normalization of inflammatory markers and resolution of liver and splenic abscess. Eradication therapy with oral co-trimoxazole and co-amoxyclav was started on the seventh week. Patient was discharged to outpatient clinic with a plan to continue combination of oral antibiotics for 12 weeks. At the end of 12 weeks she was well with complete neurological resolution and no evidence of a relapse.

**Conclusions:**

Guillaine Barre syndrome is a rare complication of melioidosis and should be suspected in a patient with melioidosis who develop lower limb weakness. Plasmapharesis can be successfully used to treat GBS associated with active melioidosis.

## Background

Melioidosis is an infection caused by the facultative intracellular gram-negative bacterium; *Burkholderia pseudomallei*. It is endemic in tropical and sub-tropical South East Asia and Northern Australia. Since the first case was reported in Sri Lanka in 1927 in a European tea broker, cases were reported sporadically [1]. However recently there had been a steady rise of reported cases [2] and now it is becoming endemic in Sri Lanka. It is not clear whether this is a true increase of incidence or an increase in diagnosis due to more awareness.

Clinical features of melioidosis are highly variable. They range from asymptomatic disease, localized skin ulcers or abscesses, acute fulminant septicaemia to chronic infection. Neurological melioidosis is very rare [3]. Although there are several reported cases of melioidosis associated with a GBS like illness [4–6] only one [4] has level 1 diagnostic certainty according to Brighton criteria (https://brightoncollaboration.org/public/resources/standards/case-definitions/main/0110/link/BC_Case%20definition_GBS.pdf).

## Case presentation

A 42 year old diabetic woman from North Western Province of Sri Lanka presented with abdominal pain, vomiting and intermittent fever for one month. She had recurrent skin abscesses 6 months before presentation which was treated with incision and drainage and a short course of antibiotics for which there was only partial improvement. Three months before presentation to us she was found to have multiple liver abscesses which were aspirated in a local hospital. The aspirate grew “coliforms” resistant to gentamicin and sensitive to ceftazidime. She was treated with cefuroxime, meropenem, co-amoxyclav and metronidazole; further identification of the organism was not done at the local hospital.

On presentation to us she had high fever and tender hepatomegaly. Her body weight was 40 kg. Rest of the examination including nervous system was normal.

Her full blood count showed leukocytosis [15.7×109/L] and granulocytosis [11.3 × 109/L], ESR was 100 mm in the 1st hour and C-reactive protein was 98.4 mg/L. Ultra sound scan of abdomen showed multiple liver and splenic abscesses.

Presence of recurrent multiple liver and splenic abscesses in a diabetic patient who had recurrent skin abscess in the past made us consider melioidosis in the differential diagnosis. Intravenous meropenem 2g eight hourly was started empirically and aspirate was sent with a sample of serum to Faculty of Medicine, University of Colombo which serves as the National Reference Laboratory, to confirm the diagnosis. There the aspirate yielded pinpoint colonies on blood, chocolate and Macconkey agar after overnight incubation. Since biochemical panels are not very accurate in diagnosis of *Burkholderia pseudomallei,* isolate was subjected to PCR which confirmed the diagnosis.

Serum antibodies to melioidin antigen using an in-house indirect haemagglutination (IHA) test based on that described by Alexander et al*.* with antigen prepared from local strains of *B.pseudomallei* [7] were positive at a titre of 1:10240.

After confirmation of melioidosis intravenous meropenem was continued. However after a week of treatment the disease course was complicated by bilateral lower limb weakness which progressed over the next 4 days. Her lower limbs were flaccid and deep tendon reflexes were absent. There was no sensory sign or bladder/bowel involvement. Cerebro-spinal fluid analysis showed protein of 114.5 mg/dl, glucose 62 mg/dl, white cells <10 mm9/L and red cells < 10 mm9/L. MRI scan of the brain was normal. The nerve conduction study showed evidence of severe peripheral neuropathy with axonal degeneration; compatible with a primary axonal type inflammatory neuropathy such as acute motor axonal neuropathy or acute sensory motor axonal neuropathy.

A diagnosis of Guillan-Barre syndrome was made based on Brighton case definition (Table 1) (https://brightoncollaboration.org/public/resources/standards/case-definitions/main/0110/link/BC_Case%20definition_GBS.pdf) and she was treated with 4 cycles of plasmapharesis on alternate days for which there was marked improvement of neurological deficit.

**Table 1:** Key diagnostic criteria and Brighton case definitions for Guillain-Barre’ syndrome [9]

Diagnostic criteria	Level of diagnostic certainty		
	1	2	3
Bilateral and flaccid weakness of limbs	+	+	+
Decreased or absent deep tendon reflexes in weak limbs	+	+	+
Monophasic course and time between onset-nadir 12 h to 28 days	+	+	+
CSF cell count <50/ml	+	+/−^a^	-
CSF protein concentration > normal value	+	+/−^a^	-
NCS findings consistent with one of the subtypes of GBS	+	+/−	-
Absence of alternative diagnosis for weakness	+	+	+

*NCS* nerve conduction studies, *CSF* Cerebro-spinal fluid

^a^If CSF is not collected or results not available, nerve electrophysiology results must be consistent with the diagnosis Guillain-Barre’ syndrome

With intravenous meropenem patient further improved and her ESR and CRP became normal. Repeat ultrasound scan of abdomen showed reduction of the size of liver and splenic abscesses.

Eradication therapy with cotrimoxazole 1920 mg 12 hourly and co-amoxyclav 625 mg 8 hourly was started on the seventh week and overlapped with intravenous antibiotic for further two weeks. Patient was discharged to outpatient clinic after two weeks of oral antibiotics with a plan to continue them for12 weeks. At the end of 12 weeks she had no residual neurological weakness and there was no sign of a relapse.

## Discussion

*Burkholderia pseudomallei*, which causes melioidosis commonly presents with superficial or deep seated abscess formation; similar to our patient. Neurological complications of melioidosis are rare. A 20 year prospective study of melioidosis conducted in Northern Australia recorded only 14 cases of neurological melioidosis out of total 540 [3]. The most common neurological manifestation was meningo-encephalitis (10 cases) [3].

Guillain-Barré syndrome (GBS) is described as a clinical entity that manifest with rapidly evolving symmetrical limb weakness, loss of tendon reflexes, absent or mild sensory signs and variable autonomic dysfunction [8]. Diagnosis of GBS is based on clinical characteristics supported by investigation findings. In 2011 Brighton collaboration which is a collaboration sponsored by the World Health Organization has developed a set of case definitions to diagnose GBS based on clinical and investigation findings (Table 1) [9] (https://brightoncollaboration.org/public/resources/standards/case-definitions/main/0110/link/BC_Case%20definition_GBS.pdf) Brighton criteria categorizes GBS into three levels of diagnostic certainty. Level 1, the highest level of diagnostic certainty, is where all criteria are present. In level 3 there are only clinical criteria with absence of an alternative diagnosis. Our patient fulfilled all clinical and investigation criteria enabling diagnosis of GBS at level 1 certainty. The 20 year prospective Darwin study reports no case of GBS in their 14 cases of neurological melioidosis. Krovvidi et.al report a case of GBS [4] where a patient had ascending paralysis, areflexia, protein-cell dissociation in CSF and demyelinating polyradiculoneuropathy who later succumbed to septic shock. Blood cultures of the patient later grew *B.pseudomallei*. Literature survey revealed 8 more cases of melioidosis where a GBS like syndrome was associated [5, 6] but none had level 1 diagnostic certainty. Therefore, this is the first case of confirmed GBS associated with melioidosis which completely recovered with treatment.

Intravenous immunoglobulin and plasmapheresis are both equally effective in GBS. [10] However because of the ease of administration intravenous immunoglobulin is currently the preferred treatment. [10] As our patient had active melioidosis when GBS was diagnosed we did not use immunoglobulin which is an immunomodulator. She recovered fully with plasmapheresis.

Detection of lower limb weakness in a patient already weak and bedbound from long standing melioidosis was very difficult. However frequent thorough assessment of the patient enabled us to actively suspect, diagnose and treat GBS early. Prompt aggressive treatment of the patient resulted in complete recovery of this axonal type of GBS which usually has a poorer prognosis than the demyelinating type. [8]

As our patient had severe melioidosis we used high dose intravenous meropenem as induction therapy based on recommendations by Timothy J.J. Inglis [11]. Induction therapy was followed by eradication therapy with oral co-trimoxazole and co- amoxyclav. Patient completely improved with this treatment and tolerated the antibiotics well. At the end of 12 weeks patient was well with no recurrence; similar to our previous experience of treatment of melioidosis.

Although our patient had a history very suggestive of melioidosis with recurrent superficial and deep abscesses, there was a delay in diagnosis resulting in significant morbidity. The isolate from liver abscess aspirate from the local hospital which was reported as “coliforms” was probably *B. psudomallei*. It should be noted that laboratories unfamiliar with this bacterium may misidentify it as “coliforms” or “pseudomonas species”. Melioidosis should always be suspected if the culture is resistant to gentamicin and other aminoglycosides but sensitive to ceftazidime especially in a diabetic patient. This will expedite the diagnosis leading to prompt treatment with excellent prognosis.

## Conclusions

Melioidosis should always be suspected in a patient with superficial or deep seated abscess formation. If pus culture yields a growth of “coliforms” or “pseudomonas species”; resistant to gentamicin and other aminoglycosides but sensitive to ceftazidime; melioidosis is very likely. As melioidosis needs treatment with a prolonged course of antibiotics, confirmation of diagnosis by PCR from a reference laboratory is necessary.

Guillaine Barre syndrome can complicate melioidosis and should be suspected in a patient with lower limb weakness. Plasmapheresis seems to be an effective method of treatment of GBS associated with melioidosis.

This case illustrates that intravenous meropenem in inductions stage followed by co-trimoxazole and co-amoxyclav in eradication stage is effective in treatment of severe and complicated melioidosis.

## Abbreviations

CRP, C-reactive protein; ESR, erythrocyte sedimentation rate; GBS, guillaine barre syndrome; IHA, indirect haemagglutination test; PCR, polymerase chain reaction; WBC, white blood count
